# Associations between Intrinsic Heart Rate, P Wave and QT Interval Durations and Pulse Wave Analysis in Patients with Hypertension and High Normal Blood Pressure

**DOI:** 10.3390/ijerph17124350

**Published:** 2020-06-17

**Authors:** Ioana Mozos, Cristina Gug, Costin Mozos, Dana Stoian, Marius Pricop, Daniela Jianu

**Affiliations:** 1 Department of Functional Sciences, “Victor Babes” University of Medicine and Pharmacy, 300173 Timisoara, Romania; ioanamozos@umft.ro; 2 Center for Translational Research and Systems Medicine, “Victor Babes” University of Medicine and Pharmacy, 300173 Timisoara, Romania; 3 Department of Microscopic Morphology, “Victor Babes” University of Medicine and Pharmacy, 300041 Timisoara, Romania; 4 Faculty of Medicine, “Victor Babes” University of Medicine and Pharmacy, 300042 Timisoara, Romania; costin_mozos@yahoo.com; 5 2nd Department of Internal Medicine, “Victor Babes” University of Medicine and Pharmacy, 300723 Timisoara, Romania; stoian.dana@umft.ro; 6 Discipline of Maxillofacial Surgery, Faculty of Dentistry, “Victor Babes” University of Medicine and Pharmacy Timisoara, 300062 Timisoara, Romania; 7 1st Department of Internal Medicine, “Victor Babes” University of Medicine and Pharmacy, 300041 Timisoara, Romania; jianu@doctor.com; 8 Department of Internal Medicine, Military Hospital, 300041 Timisoara, Romania

**Keywords:** pulse wave velocity, heart rate, intrinsic heart rate, QT interval, P wave, hypertension, high normal blood pressure, electrocardiogram, early vascular aging

## Abstract

The present study aimed to explore the relationship between electrocardiographic (ECG) and pulse wave analysis variables in patients with hypertension (HT) and high normal blood pressure (HNBP). A total of 56 consecutive, middle-aged hypertensive and HNBP patients underwent pulse wave analysis and standard 12-lead ECG. Pulse wave velocity (PWV), heart rate, intrinsic heart rate (IHR), P wave and QT interval durations were as follows: 7.26 ± 0.69 m/s, 69 ± 11 beats/minute, 91 ± 3 beats/minute, 105 ± 22 mm and 409 ± 64 mm, respectively. Significant correlations were obtained between PWV and IHR and P wave duration, respectively, between early vascular aging (EVA) and P wave and QT interval durations, respectively. Linear regression analysis revealed significant associations between ECG and pulse wave analysis variables but multiple regression analysis revealed only IHR as an independent predictor of PWV, even after adjusting for blood pressure variables and therapy. Receiver-operating characteristic (ROC) curve analysis revealed P wave duration (area under curve (AUC) = 0.731; 95% CI: 0.569–0.893) as a predictor of pathological PWV, and P wave and QT interval durations were found as sensitive and specific predictors of EVA. ECG provides information about PWV and EVA in patients with HT and HNBP. IHR and P wave durations are independent predictors of PWV, and P wave and QT interval may predict EVA.

## 1. Introduction

Central and Eastern Europe are regions with a high cardiovascular burden [1]. Romania was considered a high-cardiovascular-risk country according to the European Guidelines [2]. Thus, prophylactic measures deserve special attention, as well as information beyond standard cardiovascular risk factors in early prediction of future cardiovascular events [3].

Arterial stiffness, the consequence of arteriosclerosis and subclinical atherosclerosis, is involved in cardiovascular disease development and, if assessed, can improve risk prediction, especially in hypertensive and intermediate risk patients [4,5,6,7,8].

Heart rate and electrocardiography, especially repolarization variables, including QT interval duration, can also provide useful information in cardiovascular risk assessment [9,10,11,12,13]. The links between increased resting heart rate and mortality include sympathetic overactivity, increased metabolic rate and systemic inflammation, present in several systemic conditions, as well as cardiovascular risk factors [12]. An accelerated heart rate enables low, oscillatory endothelial shear stress and increased tensile stress, inducing a proinflammatory phenotype, with elevated levels of adhesion molecules, promoting atherogenesis and arterial stiffness [14,15]. Intrinsic heart rate is the heart rate under the simultaneous beta-blockade and muscarinic receptor blockade with propranolol and atropine, respectively [16,17].

Chronological age has some limitations when assessing cardiovascular risk [18]. Vascular age can be assessed considering arterial stiffness, and early vascular aging can be defined if one’s vascular age increases faster than one’s chronological age, related to the upper 10, 20 or 25% of the pulse wave velocity (PWV) distribution in the population [19].

Arterial stiffness is characterized by a decrease of elastin content, as well as by an increased production and accumulation of collagen within the arterial wall [20]. Arterial stiffness, assessed by pulse wave analysis, especially pulse wave velocity, is increased in hypertensive patients, regardless of blood pressure levels, and is related to endothelial dysfunction, vascular remodeling and progression of blood pressure [21,22]. On the other hand, increased arterial stiffness has a major effect on systolic blood pressure, wave reflections and cardiovascular risk [22].

Considering the importance of prophylactic measures in hypertensive patients and the limits of the classical cardiovascular risk factors in predicting cardiovascular events, new, simple, inexpensive surrogate biomarkers are needed. Our objective is to explore the relationship between electrocardiographic (ECG) and pulse wave analysis variables in patients with hypertension and high normal blood pressure. We hypothesized that ECG variables can reveal increased arterial stiffness and early vascular aging in patients with elevated blood pressure values.

## 2. Materials and Methods

### 2.1. Study Population

The study included 56 consecutive hypertensive and high normal blood pressure (HNBP) patients, recruited from the Military Hospital Timisoara in the period of July 2016–March 2017.

Patients with essential hypertension and high normal blood pressure, aged between 18 and 55 years, were included. Essential hypertension and high normal blood pressure were diagnosed according to the criteria of the European Society of Cardiology [23]. The most important exclusion criteria were secondary hypertension, atrial fibrillation, diabetes mellitus, history of coronary heart disease, myocardial infarction, stroke, transient ischemic attack or peripheral arterial disease, systemic inflammatory processes, active infections, trauma and therapy with statins [24].

The investigations were performed considering the principles outlined in the Declaration of Helsinki [25]. The study was approved by the “Victor Babes” University of Medicine and Pharmacy (2743/9 March 2016). Written informed consent was obtained from each study participant and the aims, procedures and implications of the study were explained to all of them.

The patients underwent pulse wave analysis and standard 12-lead electrocardiogram (ECG). Additional data related to diagnosis and therapy of the patients were available from medical records. The investigations were performed independently by different skilled investigators, who were not aware of the patient’s diagnosis and data.

### 2.2. Mobil-O-Graph

Pulse wave analysis, including pulse wave velocity (PWV), augmentation index and pressure (AI, AP), vascular age and central and peripheral blood pressure were assessed using a Mobil-O-Graph (IEM GmbH, Stolberg, Germany), a noninvasive, cuff-based, one site, validated device. Pulse wave velocity is the speed at which the arterial pulse wave propagates along an arterial segment [26]. Augmentation pressure (AP) is the increment in aortic pressure above its first systolic shoulder [27]. Augmentation index is the ratio between augmentation pressure and pulse pressure, a measure of the contribution of wave reflection on the arterial pressure waveform [28]. The methodology and the significance of the variables were previously published [24]. PWV and vascular age were estimated from the reconstructed aortic pulse waveform via mathematical models [29]. Early vascular aging was considered when one’s vascular age was higher than the chronological age.

### 2.3. Standard 12-Lead ECG

Standard ECG was recorded at a paper speed of 25 mm/s. Resting heart rate (HR), P wave (P), PR (PR—the time from the onset of the P wave to the start of the QRS complex) and QT interval duration (QT) were automatically measured. QT was corrected considering heart rate, according to the Bazett formula, resulting in heart rate corrected QT interval (QTc). Intrinsic heart rate (IHR = 118 − (0.57 × age)) and the difference between IHR and resting heart rate (DHR) were also calculated [16]. The amplitude of the R wave in leads V5 (RV5) and V6 (RV6) and of the S wave in leads V1 (SV1) and V2 (SV2) were manually measured.

### 2.4. Statistical Analysis

Categorical data are given as numbers and percentages, continuous data as means ± standard deviation. Correlations (Bravais–Pearson’s, Kendall’s and Spearman’s), univariate and multivariate linear regression analysis and receiver-operating characteristic curve (ROC) analysis were used as statistical methods. Analyses were performed using IBM SPSS base edition (IBM, Armonk, NY, USA). Normality testing of dependent variables was performed using Q–Q plots, skewness and kurtosis. A *p* < 0.05 was considered statistically significant. PASS 2019 (NCSS Statistical Software, Kaysville, UT, USA) was used to conduct a power analysis before the study to calculate the sample size needed for the present study [24].

## 3. Results

The study included middle-aged patients (48 ± 6 years) with hypertension and high normal blood pressure, most of them male (57%) [24]. Table 1 includes demographic data, the values measured or calculated for several electrocardiographic variables and pulse wave analysis results. A total of 21 patients (37.5%) had high normal blood pressure, 16 (28.6%) grade 1, 13 (23.2%) grade 2 and 6 (10.7%) grade 3 hypertension. Hypertension grades considered the guidelines of the European Society of Cardiology for the management of arterial hypertension [23]. Therapy included angiotensin converting enzyme inhibitors, sartans, diuretics, beta blockers and calcium channel blockers.

Normality testing of dependent variables using Q–Q plots, skewness and kurtosis revealed that the data were approximately normally distributed.

Correlations were calculated for all ECG and pulse wave analysis variables. Significant correlations were obtained just between pulse wave velocity (PWV) and intrinsic heart rate (IHR) and the difference between IHR and resting heart rate (DHR) (Table 2). P wave duration significantly correlated with augmentation pressure, and augmentation index with heart rate corrected QT interval duration (QTc) and the amplitude of the S wave in lead V1 (SV1), respectively. No significant correlations were obtained between PWV and resting heart rate.

Linear regression analysis revealed significant associations of IHR with PWV and early vascular aging (EVA). A prolonged QTc interval (exceeding 450 ms: QTc450) was significantly associated with EVA and augmentation index (AI). The association EVA-long QTc lost its significance after adjusting for systolic blood pressure (SBP). Significant associations were also found between AI and S wave in V1 (Table 3).

Multiple linear regression analysis revealed only IHR as an independent predictor of PWV, even after adjusting for blood pressure variables and heart rate and blood pressure-lowering therapy (Table 4). IHR lost its significant association with EVA after adjusting for blood pressure variables (SBP, mean arterial pressure (MAP) and pulse pressure (PP)) and therapy.

**Figure 1:**
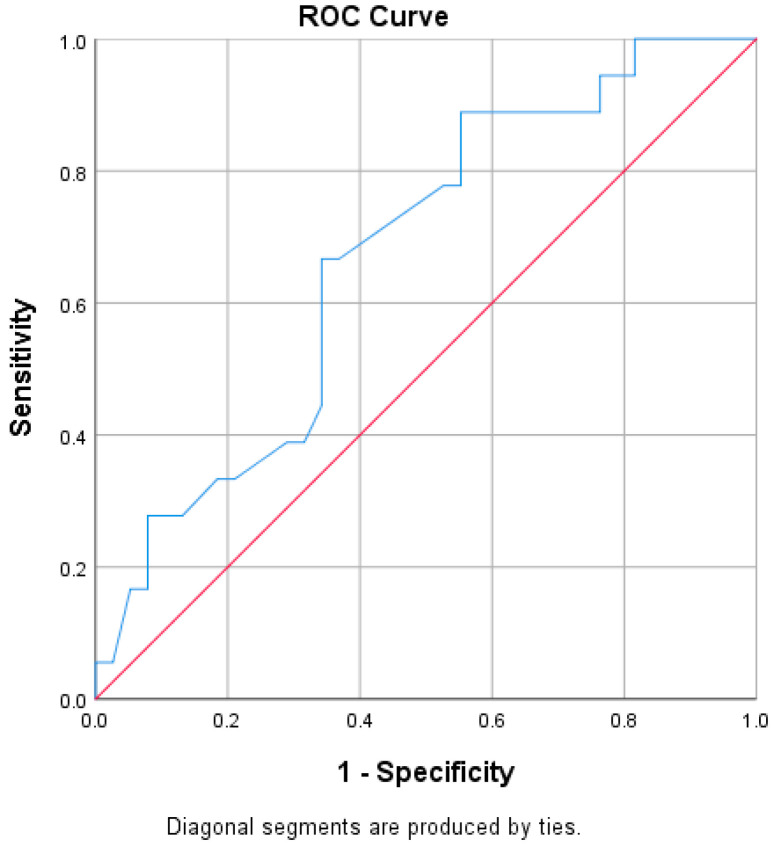
QT interval as a sensitive and specific predictor of early vascular aging (EVA) (AUC = 0.671; 95% CI: 0.525–0.817), *p* = 0.04.

ROC curve analysis revealed P wave duration as a predictor of pathological pulse wave velocity (pPWV) (Table 5). P wave and QT interval durations (Figure 1) were found as sensitive and specific predictors of EVA.

## 4. Discussion

The present study reveals significant correlations and associations between electrocardiographic biomarkers and pulse wave velocity and early vascular aging, respectively, in middle-aged patients with hypertension and high normal blood pressure. Intrinsic heart rate and P wave duration were found as independent predictors of pulse wave velocity, while QT interval and P wave durations as independent predictors of early vascular aging, confirming our hypothesis that ECG variables can reveal increased arterial stiffness and early vascular aging in patients with elevated blood pressure values.

The influence of heart rate on PWV measurements remains controversial, with conflicting results observed in both acute and epidemiological studies [30,31,32,33]. A significant blood pressure independent association between heart rate and PWV was mentioned [33], significant only for subjects with increased aortic stiffness [34]. A higher resting heart rate was independently associated with arterial stiffness in healthy Korean adults [35]. Heart rate plays differential roles in the development of arterial stiffness and subclinical atherosclerosis during young adulthood [31]. Heart rate related changes in arterial stiffness were attributed to the viscoelasticity of the arterial wall [33]. According to other authors, the increase of arterial stiffness with heart rate is considered as an adjustment of the arterial tree to enable optimization of its performance and is more important in arteries supplying end capillaries with high permeability and low reflection coefficients [36]. A lower heart rate prolongs ejection time, increases overlapping of the forward and backward pressure waves by widening the forward arterial waveform [37]. The present study does not reveal any relationship between resting heart rate and pulse wave velocity and early vascular aging but reports significant correlations and associations between intrinsic heart rate and PWV and EVA, respectively. Multiple regression analysis demonstrates the independent predictive value of IHR for PWV, even after controlling for blood pressure and therapy—variables that influence arterial stiffness. According to our knowledge, this is the first study reporting a relationship between intrinsic heart rate and arterial stiffness. The possible link between IHR and arterial stiffness could be, besides age, also exercise training, considering that IHR is lower with training [17]. Training-induced bradycardia is related to intrinsic electrophysiological changes in the sinus node, with downregulation of ion channels, especially funny channel HCN4 [38]. On the other hand, training may also improve arterial stiffness [39].

Several other studies reported links between electrocardiographic and pulse wave analysis biomarkers. QTc interval, prolonged in patients with Systemic Lupus Erythematosus, was related to subclinical atherosclerosis, measured by carotid-femoral pulse-wave velocity, after controlling for age and hypertension [40]. A study including 54 apparently healthy participants revealed significant correlations and associations between Tpeak–Tend interval, a measurement of dispersion of the terminal part of the repolarization and predictor of life-threatening ventricular arrhythmias, and both brachial and aortic augmentation index, pulse wave velocity and early vascular aging [41]. The Nagahama Study Group reported the longer corrected QT interval as an independent determinant of increased augmentation index and smaller pulse pressure amplification in persons free from cardiovascular symptoms and not receiving insulin therapy [37]. The possible mechanisms explaining the association of impaired pulse wave and repolarization variables are electrophysiological remodeling related to aging, including action potential prolongation, blunted ionic exchanges across sarcolemma, increased myocardial fibrosis, increased ventricular load or subendocardial ischemia due to microvascular coronary atherosclerosis [42,43,44]. A longer ventricular conduction period broadens the forward arterial pressure wave and increases the overlapping of the forward and backward pressure wave, which might explain the elevated augmentation index, which is also a marker of arterial stiffness, in patients with a long QT interval [37]. The present study reveals the QT interval as a sensitive and specific predictor of EVA.

P wave duration was previously correlated with arterial stiffness variables, related to interatrial block, in overweight patients [45]. Body mass index was 27.14 ± 6.01 kg/m^2^, with a high prevalence of overweight (25%) and obesity (25%), which might be associated with a sedentary lifestyle in our study, but P wave duration exceeded 120 ms only in 7 patients (13%). The possible link could also be left atrial volume. A significant relationship between carotid arterial stiffness and left atrial volume was reported previously in patients with untreated hypertension [46]. Ruaengsri et al. demonstrated in a canine model of mitral regurgitation that an increase of left atrial size is associated not only with an increased atrial fibrillation inducibility but also with a decrease in left ventricular ejection fraction, with an increase of end-systolic and end-diastolic volume [47]. A study using the Campania Salute Network registry demonstrated that left atrial size was an independent predictor of cardiovascular events, regardless of other coexisting signs of target organ damage [48]. Other factors that must be considered in the aging heart include atrial fibrosis associated with atrial conduction slowing and atrial fibrillation [49]. Our study reveals P wave duration as an independent predictor of both EVA and pathological PWV.

Study limitations: arterial stiffness and early vascular aging are surrogate endpoints for cardiovascular events but using them demonstrates the prophylactic character of our study. The cross-sectional study design does not assure causality between the evaluated variables. The sample size is relatively small but the power analysis demonstrated an appropriate number of patients. Another limitation is related to the selection of study participants, including hypertensive patients receiving antihypertensive and heart rate-lowering drugs, which may question the validity of some results. However, the results did not change after controlling for blood pressure and heart rate-lowering therapy in the multiple regression analysis. The study lacks echocardiographic parameters and information about physical activity, which were not available for the patients included in the study but will be the aim of a future study. Mobil-O-Graph estimates pulse waves using an oscillometric method, which differs from the most widely applied applanation tonometry. Previous validation studies in different study populations, including hypertensive patients, revealed acceptable agreement between Mobil-O-Graph derived variables and invasive and noninvasive measurements and a good reproducibility of the technique [50,51,52].

Strengths of the study lie in the recruitment of real-world, middle-aged participants with grade 1–3 hypertension and high normal blood pressure, with a PWV increased for age in 21.4% of the participants and with both high and low cardiovascular risk, providing insights in terms of heart-vascular coupling rather than substitution of arterial stiffness measurement by ECG technique. As far as we know, our study is the first one reporting a significant relationship between intrinsic heart rate and arterial stiffness, respectively, as well as between P wave duration and EVA. The clinical implications of the present study are related to the prognostic importance of increased arterial stiffness and early arterial aging for cardiovascular risk. Simple, cost-effective, reproducible, quick to determine biomarkers, such as intrinsic heart rate, an index of sinus node function regardless of autonomic control, the duration of the P wave and QT interval can provide valuable, noninvasive information related to large vessels, arterial age and cardiovascular risk stratification in treated hypertensive patients, enabling personalized diagnosis and treatment of each patient, providing further targets for strategies to improve clinical outcomes and enable selection of patients requiring more aggressive therapy. Larger studies are needed in hypertensive patients to confirm the findings of the present study, to demonstrate that aging related functional and structural changes in the heart and artery are simultaneous and to reveal any connection between interstitial fibrosis related to aging associated remodeling of the myocardium, collagen content in the sinus node and arterial wall. Further prospective studies must confirm the synergistic prognostic effect of pulse wave velocity, early vascular aging, intrinsic heart rate, P wave and QT interval durations in hypertensive patients.

## 5. Conclusions

ECG provides information about pulse wave velocity and early vascular aging in patients with hypertension and high normal blood pressure. IHR and P wave duration are independent predictors of pulse wave velocity in patients with hypertension and high normal blood pressure. P wave and QT interval duration are sensitive and specific predictors of EVA.

## Figures and Tables

**Table 1:** Characteristics of the study population.

Variable	Mean ± SD
Age (years)	48 ± 6 years
Male	32 (57%)
High normal blood pressure	12 (37.5%)
Grade 1 Hypertension	16 (28.6%)
Grade 2 Hypertension	13 (23.2%)
Grade 3 Hypertension	6 (10.7%)
SBP (mmHg)	137 ± 14
DBP (mmHg)	91 ± 11
MAP (mmHg)	111 ± 11
PP (mmHg)	45 ± 10
PVW (m/s)	7.26 ± 0.69
AP (mmHg)	7.9 ± 6.5
AI (%)	20 ± 13.71
EVA (%)	18 patients (32.1)
HR (beats/min)	69 ± 11
IHR (beats/min)	91 ± 3
DHR (beats/min)	22 ± 11
P (ms)	105 ± 22
PR (ms)	147 ± 31
QRS (ms)	95 ± 17
QT (ms)	409 ± 64
QTc (ms)	434 ± 68
QTc > 450 ms	14 patients (25%)
RV5 (mm)	11 ± 6
RV6 (mm)	11 ± 5
SV1 (mm)	7 ± 4
SV2 (mm)	8 ± 3
Therapy	angiotensin converting enzyme inhibitors (30%)
	sartans (17.5%)
	diuretics (32.5%)
	beta blockers (32.5%)
	calcium channel blockers (7.5%)

SBP—systolic blood pressure; DBP—diastolic blood pressure; MAP—mean arterial pressure; PP—pulse pressure; PVW—pulse wave velocity; AP—augmentation pressure; AI—augmentation index; EVA—early vascular aging; HR—resting heart rate; IHR—intrinsic heart rate; DHR—the difference between IHR and resting heart rate; P—P wave duration; PR—PR interval duration; QRS—duration of the QRS complex; QT—duration of the QT interval; QTc—heart rate corrected QT interval using the Bazett formula; RV5—amplitude of the R wave in lead V5; RV6—amplitude of the R wave in lead V6; SV1—amplitude of the S wave in lead V1; SV2—amplitude of the S wave in lead V2.

**Table 2:** Correlations between electrocardiographic and pulse wave analysis variables.

Correlated Variables	Correlation Coefficient	*p*
PWV−IHR	rP = −0.556	˂0.0001
	rK = −0.349	˂0.0001
	rS = −0.467	˂0.0001
PWV−DHR	rP = −0.271	0.044
	rK = −0.228	0.015
	rS = −0.311	0.020
PWV−HR	rP = 0.111	0.416
AP−HR	rP = −0.157	0.249
AP−P	rK = −0.206	0.025
	rS = −0.275	0.040
AP−SV1	rK = −0.230	0.019
	rS = −0.305	0.022
AI−QTc	rS = −0.274	0.041
AI−SV1	rP = −0.286	0.033
	rK = −0.212	0.027
	rS = −0.295	0.027

rP—Bravais−Pearson’s correlation coefficient; rK—Kendall’s correlation; rS—Spearman’s correlation; PWV—pulse wave velocity; AI—augmentation index, AP—augmentation pressure, IHR—intrinsic heart rate, DHR—the difference between IHR and resting heart rate; P—P wave duration; QT—duration of the QT interval; QTc—heart rate corrected QT interval using the Bazett formula; SV1—amplitude of the S wave in lead V1.

**Table 3:** Electrocardiographic predictors of pulse wave velocity (PWV) and early vascular aging (EVA). Results of linear regression analysis. Covariates-free analysis.

Predicted Variable	Predictor	Unst. Beta	Stand. Beta	Signific.	95% CI	R^2^	Adj. R^2^
PWV	IHR	−0.119	−0.556	<0.001	−0.167–−0.07	0.556	0.309
EVA	IHR	0.038	0.285	0.047	0.001–0.075	0.081	0.062
EVA	QTc450	−0.333	−0.309	0.020	−0.613–−0.053	0.309	0.096
AI	SV1	−1.012	−0.286	0.033	−1.937–−0.086	0.286	0.082
AI	QTc450	−8.286	−0.264	0.049	−16.542–−0.030	0.264	0.070

IHR—intrinsic heart rate; QTc450—heart rate corrected QT interval duration exceeding 450 ms; AI—augmentation index; SV1—S wave amplitude in lead V1; Unst. Beta—Unstandardized Beta; Stand. Beta—Standardized Coefficients Beta; Signific.—significance; R^2^—R squared; Adj. R^2^—adjusted R squared.

**Table 4:** Results of multiple linear regression analysis.

Predicted Variable	Predictor	Unst. Beta	Stand. Beta	*p*	95% CI	R^2^	Adj. R^2^	Signific.
PWV	IHR adjusted for SBP, MAP	−0.133	−0.623	˂0.001	−0.177–−0.089	0.476	0.446	˂0.001
PWV	IHR adjusted for SBP, MAP, therapy	−0.133	−0.620	˂0.001	−0.177–−0.088	0.479	0.438	˂0.001
EVA	IHR adjusted for SBP, MAP, PP	0.021	0.147	0.220	−0.013–−0.056	0.309	0.252	0.001
EVA	IHR, therapy	0.028	0.192	0.157	−0.011–−0.067	0.053	0.017	0.239

PVW—pulse wave velocity; EVA—early vascular aging; IHR—intrinsic heart rate; SBP—systolic blood pressure; MAP—mean arterial pressure; PP—pulse pressure; Unst. Beta—Unstandardized Beta; Stand. Beta—Standardized Coefficients Beta; Signific—significance; R^2^—R squared; Adj. R^2^—adjusted R squared; therapy—therapy with beta-blockers, calcium-channel blockers, sartans and angiotensin converting enzyme inhibitors.

**Table 5:** Results of receiver-operating characteristic (ROC) curve analysis for early vascular aging (EVA) and pathological pulse wave velocity (pPWV).

Test Variable	State Variable	AUC (95% CI)	*p*	Cut-Off Value	Sensitivity	Specificity
pPWV	P	0.731 (0.569–0.893)	0.015	94.5	83.3%	70.5%
EVA	P	0.675 (0.527–0.824)	0.035	94.5	66.7%	71.1%
EVA	PR	0.622 (0.458–0.786)	0.143			
EVA	QT	0.671 (0.525–0.817)	0.04	393.5	66.7%	65.8%
EVA	QTc	0.659 (0.52–0.799)	0.056			
EVA	RV5	0.605 (0.428–0.783)	0.207			

EVA = early vascular aging; PWV = pulse wave velocity; pPWV = pathological pulse wave velocity (increased for age); P = P wave duration; PR = PR interval duration; QT = QT interval duration; QTc = heart rate corrected QT interval using the Bazett formula; RV5 = amplitude of the R wave in lead V5; AUC = area under the curve; CI = confidence interval.
